# Prognostic significance of interim PET/CT based on visual, SUV-based, and MTV-based assessment in the treatment of peripheral T-cell lymphoma

**DOI:** 10.1186/s12885-015-1193-1

**Published:** 2015-03-28

**Authors:** Sung-Hoon Jung, Jae-Sook Ahn, Yeo-Kyeoung Kim, Sun-Seog Kweon, Jung-Joon Min, Hee-Seung Bom, Hyeoung-Joon Kim, Yee Soo Chae, Joon Ho Moon, Sang Kyun Sohn, Sang Woo Lee, Byung Hyun Byun, Young Rok Do, Je-Jung Lee, Deok-Hwan Yang

**Affiliations:** 1 Department of Hematology-Oncology, Chonnam National University Hwasun Hospital, 322 Seoyangro, Hwasun, Jeollanamdo 519-763 Republic of Korea; 2 Institute for Molecular Photonic Imaging Research, Chonnam National University Hwasun Hospital, Hwasun, Jeollanam-do Republic of Korea; 3 Department of preventive Medicine, Chonnam National University Medical School, Gwangju, Republic of Korea; 4 Department of Hematology/oncology, Kyungpook National University Hospital, Daegu, Republic of Korea; 5 Department of Nuclear Medicine, Kyungpook National University Hospital, Daegu, Republic of Korea; 6 Nuclear Medicine, Korean Cancer Center Hospital, Seoul, Republic of Korea; 7 Hemato-oncology, Keimyung University Dongsan medical center, Daegu, Korea

**Keywords:** Peripheral T-cell lymphoma, Positron emission tomography, Interim PET/CT, Prognosis, Prognostic model

## Abstract

**Backgrounds:**

The role of interim PET/CT in peripheral T-cell lymphoma (PTCL) is less identified compared to other subtype of lymphoma. This study prospectively investigated the prognostic accuracy of sequential interim PET/CT using visual and quantitative assessment to determine whether it provided prognostic information for the treatment of PTCL.

**Methods:**

Sixty-three patients with newly diagnosed PTCL were enrolled, and 59 patients underwent interim PET/CT after three or four courses of induction treatment. The response of interim PET/CT was assessed by three parameters: the Deauville five-point scale (5-PS), ΔSUVmax, and ΔMTV2.5.

**Results:**

Over a median follow up of 40.3 months, each assessment of interim PET/CT using the 5-PS, ΔSUVmax, and ΔMTV2.5 had predictive value for progression-free survival. To increase the predictive accuracy of interim PET/CT, we divided patients into three groups according to the sum of scores for three adverse responses based on the visual, SUV-based and MTV-based assessment: favorable, intermediate, and poor responder. The clinical outcome of patients in the favorable group was significantly superior to patients in the poor or intermediate group.

**Conclusion:**

Visual, quantitative SUV-based, and MTV-based assessment in interim PET/CT are valuable for early treatment response assessment in patients with PTCL, and the combined approach using the three parameters was more efficient in discriminating between patients with different survival outcomes compared with single-parameter assessment.

**Trial registration:**

NCT01470066.

## Background

Peripheral T-cell lymphoma (PTCL) is a heterogeneous group of aggressive lymphomas, in which the T-cell phenotype itself is associated with unfavorable prognostic factors compared with B-cell-phenotype lymphomas [1]. Although PTCL is chemosensitive to conventional regimens, the clinical outcomes have been uniformly disappointing. To improve the therapeutic outcome, frontline high-dose chemotherapy followed by autologous stem cell transplantation (HDT/ASCT) or early therapeutic intensification based on the interim response has been presented as a rescue option from poor prognosis. However, the effect on distinct PTCL subtypes, the optimal time point of assessing the response during the clinical course, and the prognostic factors for predicting better outcomes remain unclear [2-4].

Several recent studies have evaluated treatment of patients with Hodgkin’s or Non-Hodgkin’s lymphoma using dose-dense therapeutic intensification based on assessment of interim positron emission tomography (PET) with ^18^F-fluoro-2-deoxy-D-glucose (FDG) to predict outcome [5-8]. Persistent FDG uptake during primary chemotherapy is associated with a poor prognosis and could be considered to escalate treatment strategies to avoid poor outcomes. However, such treatment modification using the interim metabolic response remains controversial, and its clinical use is not permitted, except in clinical trials. Moreover, the lack of agreed-upon standardized response criteria, difference in the percent risk according to the international prognostic index (IPI) or prognostic index of peripheral T-cell lymphoma (PIT), and different treatment modalities used have contributed to the variability of outcomes and poor reproducibility [9-11]. In an attempt to standardize the reporting criteria of interim PET/CT, the ‘International Workshop on Interim PET in Lymphoma’ suggested visual response criteria using the Deauville five-point scale (5-PS) and investigated the standardized uptake value (SUV) in comparison with this visual system [12,13]. Methods to improve the predictive value of interim PET/CT include quantitative approaches, such as measuring the rate of reduction in maximum SUV (**Δ**SUVmax) or metabolic tumor volume (**Δ**MTV), defined as the volume of tumor tissue with increased FDG uptake. A quantitative approach might be more appropriate in the early response of PET to reduce the false-positive rate or decrease the inter-observer variability in interpretation [14-16].

Data concerning the role of PET/CT in patients with PTCLs using interim assessment with FDG uptake are limited. T/NK cell lymphomas are mostly FDG avid, with higher uptake in more aggressive subtypes but lower uptake in cutaneous disease [17-19]. However, because FDG is not a tumor-specific substance, it may accumulate in various benign conditions, possibly producing false-positive results. The prognostic significance of positive interim FDG-PET/CT based on visual assessment may be reduced by tracer uptake by inflammatory or infectious lesions, particularly lymphoma at special sites or anatomical FDG uptakes in patients with nasal NK/T or with extranodal PTCLs [20,21]. Thus, the combination of visual and quantitative assessments could decrease the pitfalls of interim PET/CT interpretation.

Therefore, interim PET/CT assessed by visual, SUV-based or MTV-based parameters could enable the early identification of a poor prognosis and allow treatment intensification or early stem cell transplantation in PTCLs. In the present study, we prospectively investigated the prognostic accuracy of sequential interim PET/CT using visual and quantitative assessment to determine whether it provided prognostic information for the treatment of PTCL.

## Methods

### Patients and study design

Sixty-three patients with newly diagnosed PTCL were enrolled from September 2006 to November 2011 (ClinicalTrials.gov Identifier: NCT01470066). The histological diagnosis of PTCLs was made by hematopathologists according to the World Health Organization (WHO) classification, and all of the biopsy specimens were reviewed through immunohistochemical examination and molecular studies. PET/CT was performed at the time of diagnosis, mid-treatment and the completion of primary chemotherapy. Briefly, all of the patients had an initial CT and PET/CT at diagnosis, a subsequent interim CT and PET/CT after three or four cycles as well as at the completion of cyclophosphamide, doxorubicin, vincristine, prednisone (CHOP)/CHOP-like, or non-anthracycline-based chemotherapy. The interim response evaluation using PET/CT was performed at 1 day prior to a scheduled chemotherapy. The final response was assessed within 1 month of completing the primary chemotherapy, with follow-up restaging every 3 months during the first year, and every 6 months thereafter. All patients, except those in stage I, were treated with six or eight cycles of an anthracycline-based regimen (CHOP or CHOP-like), dose-adjusted etoposide, vincristine, doxorubicin, cyclophosphamide, and prednisone (EPOCH), VIDL (ifosphamide, etoposide, dexamethasone and L-asparaginase) or IMEP (ifosphamide, methotrexate, etoposide, and prednisolone) as induction chemotherapy. Patients in stage I were treated with four cycles of primary chemotherapy followed by involved field radiation therapy (IFRT, 30 Gy). The prognostic importance was classified according to the International Prognostic Index (IPI) and Prognostic Index for PTCL (PIT) (age, lactate dehydrogenase [LDH], performance status, and bone marrow involvement). All patients were eligible for inclusion after the protocol was approved by the Institutional Review Board of Chonnam National University Hwasun Hospital in accordance with the Declaration of Helsinki. All patients provide written informed consent before enrollment.

### ^18^F-FDG PET/CT and image analysis

All of the patients underwent ^18^F-FDG PET/CT using a Discovery ST PET/CT system (GE Healthcare), consisting of a bismuth germanate full scanner and a 16-detector-row CT scanner. The patients fasted for at least 6 h prior to the intravenous administration of ^18^F-FDG (7.4 MBq per body weight) to ensure a serum glucose level below 7.2 mmol/L. At 60 min after ^18^F-FDG administration, transmission data were acquired using low-dose CT (120 kV, automated from 10 to 130 mA, a 512 × 512 matrix, a 50-cm field of view (FOV), 3.75-mm slice thickness, and a rotation time of 0.8 s), extending from the base of the skull to the proximal thighs. Immediately after CT acquisition, PET emission scans were acquired in the same anatomic locations with a 15.7-cm axial FOV acquired in the two-dimensional mode with a 128 × 128 matrix. The CT data were used for attenuation correction. The images were reconstructed using a conventional iterative algorithm (OSEM). A workstation (AW Volume Share™) providing multi-planar reformatted images was also used for image display and analysis. The initial and final conventional CT and PET/CT were assessed according to the revised International Workshop Criteria (IWC) [22]. The PET/CT scans were read by two nuclear medicine physicians who had no knowledge of subject or clinical information.

### Response of interim PET/CT based on the three parameters of visual, SUV-based and MTV-based assessment

We first classified patients using the five-point scale (5-PS) based on the Deauville criteria on interim PET/CT analysis [12]: 1, no uptake; 2, uptake ≤ mediastinum; 3, uptake > mediastinum but ≤ liver; 4, uptake moderately increased compared with the liver uptake at any site; 5, markedly increased uptake compared with the liver at any site and new sites and/or new sites of disease. Interim PET/CT images were graded as negative or positive by comparison with initial PET/CT scans, and grades 1–3 were considered negative, and grades 4–5 were considered positive [23]. This grading process was independent of the size of the residual tumor.

Second, we classified the patients using the quantitative analysis of ^18^F-FDG uptake changes based on the percentage of SUVmax reduction between the initial and interim PET/CT scans. On axial, coronal, or sagittal coregistered PET/CT slices, simple circular regions of interest (ROIs) were placed to cover the lesion or background. SUV measurements were corrected for body weight according to the following standard formula: Mean ROI activity (MBq/ml)/[Injected dose (MBq)/Body weight (kg)] [24]. For each PET dataset, the maximum SUV (SUVmax) was defined as the highest SUV among all hypermetabolic tumor foci. The SUVmax reduction rate (**Δ**SUVmax) was calculated as follows:

$$ \Delta \mathrm{SUVmax}\ \left(\%\right) = 100 \times \left[\mathrm{SUVmax}\ \left(\mathrm{initial}\right)\ \hbox{--}\ \mathrm{SUVmax}\ \left(\mathrm{interim}\right)\right]/\mathrm{SUVmax}\ \left(\mathrm{initial}\right) $$

If all of the lesions had disappeared on interim PET, ROIs were drawn in the same area on interim PET as on baseline PET.

We finally classified patients using quantitative analysis of metabolic volume changes based on the percentage of MTV reduction (**Δ**MTV) between the initial and interim PET/CT scans. To define the exact tumor margins around the target lesions, SUV2.5 was used following previous reports [25,26], indicating that the tumor volume area in PET/CT was delineated by a circle encompassing regions with an SUV cutoff value of 2.5. MTV2.5 was measured using the AW Volume Share™ workstation (GE Healthcare) on the fused PET/CT images [27]. AW Volume Share™ allows automatic registration and fusion between two volumetric acquisitions that originate from different acquisition modalities. The active MTV2.5 was measured in a three-dimensional manner by selecting the volume of interest (VOI) on the axial image, and the VOI size was manually regulated on the corresponding coronal and sagittal images to include entire active tumors in the VOI. The SUVmax and sum of the tumor volumes in all of the hypermetabolic tumor foci were computed automatically by the program. The MTV2.5 reduction rate (**Δ**MTV2.5) was calculated by the same formula used for **Δ**SUVmax.

### Statistical analysis

Progression-free survival (PFS) was used as an endpoint to evaluate the prognostic significance of interim PET/CT. PFS was calculated from the treatment start time to the first recording of disease progression, death from any cause or loss of follow-up period. Overall survival (OS) was defined as the period from the start of treatment to the date of the last follow up or death from any cause. Patients whose disease did not progress would be censored using the date at which they were last known to show no progression. The distribution of patients for OS and PFS was estimated using the Kaplan–Meier method and were compared by the log-rank test for the association between clinical prognostic factors and the probability of treatment failure.

To evaluate the optimal cutoff value of SUVmax or the MTV2.5 reduction rate for predicting the PFS, receiver-operating characteristic (ROC) analysis was performed. A large area under the ROC curve (AUC) indicates greater predictive power for survival. An AUC less than 0.5 indicates no predictive ability, whereas an AUC greater than 0.5 represents predictive ability statistically. The multivariate Cox’s proportional-hazards model was used to analyze all influences found to be significant in the univariate analysis. Probability values less than 0.05 were deemed to indicate statistical significance, and the results were expressed as means ± standard error of the mean (SEM).

## Results

### Patients’ characteristics and outcome

**Figure 1:**
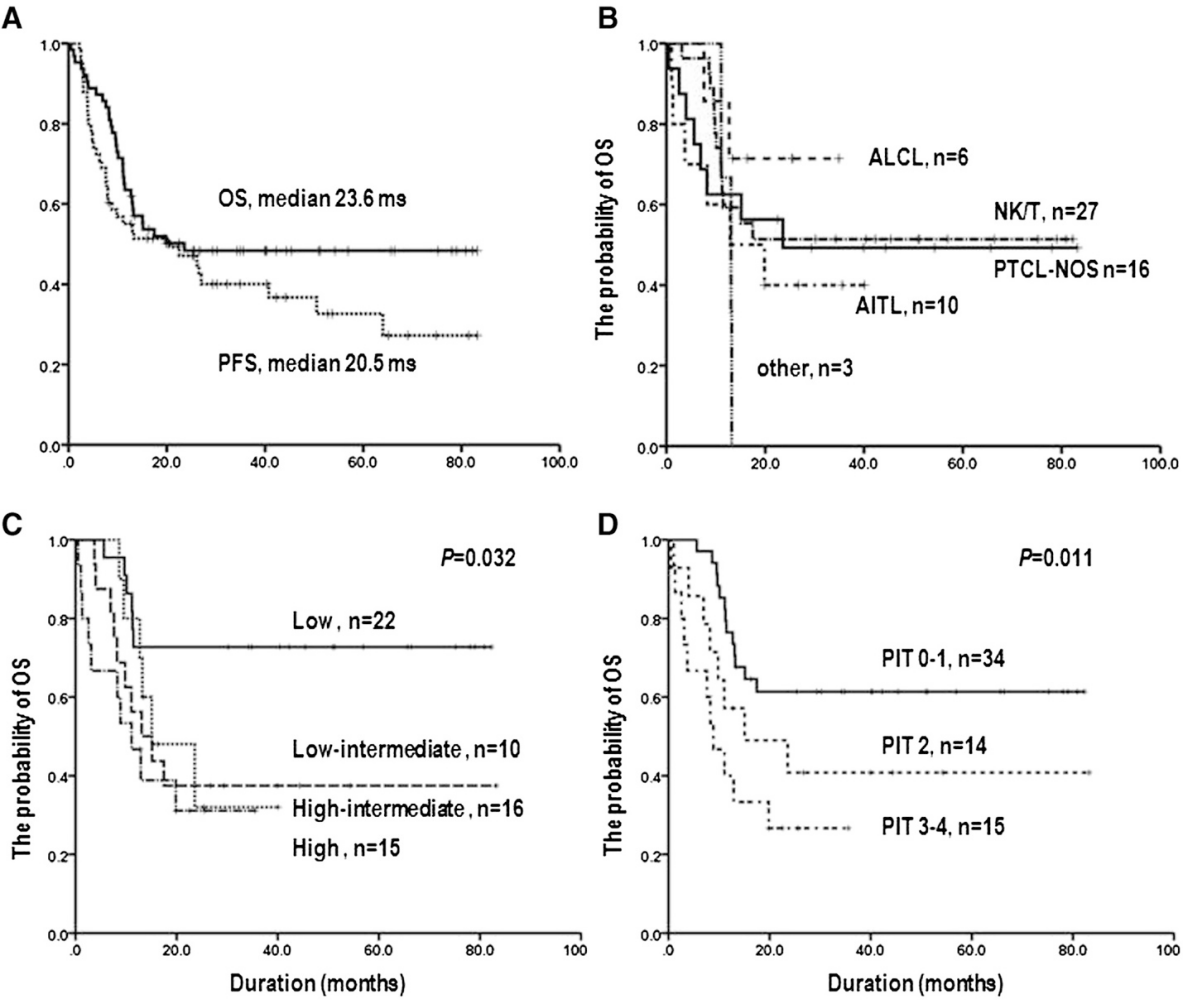
**Kaplan-Meier survival curves for progression-free survival (PFS) and overall survival (OS) in all patients with PTCL (A), and OS according to histologic subtype (B), IPI risk (C), and PIT risk (D).**

The clinical characteristics of the 63 enrolled patients are summarized in Table 1. Their median age was 60 years (range, 20–81 years) with 49.2% of patients aged more than 60 years. Thirty-nine patients (61.9%) presented with advanced-stage disease, and 18 (28.6%) had bone marrow involvement. The histological subtypes were PTCL unspecified (n = 17; 27%), angioimmunoblastic T-cell lymphoma (n = 10; 15.9%), NK/T cell (n = 27; 42.9%), and others. Thirty-one patients (49.2%) were classified as high risk based on IPI, and 29 (46%) were classified as high risk (more than 2 factors) based on PIT. Most patients (84.1%) were treated with the CHOP/CHOP-like regimen with a median number of six cycles, and 10 (15.9%) were treated with other induction chemotherapy including IMVP, EPOCH, and VIDL. Fifteen patients, primarily with NK/T cell lymphoma, received a short course of chemotherapy followed by IFRT. Fifty-nine patients underwent interim PET/CT based on the three parameters. Twenty-four patients (38.1%) were classified as having a positive metabolic uptake (grade 4 and 5) based on the visual assessment using 5-PS. According to the response after primary chemotherapy, 34 (54.0%) patients achieved a complete response (CR), 7 (11.1%) achieved a partial response (PR), and 18 (28.6%) showed stable disease (SD) or progression. Nine (14.3%) patients, including those with PR after primary chemotherapy or with high-risk factors, underwent autologous stem cell transplantation as a frontline consolidation. Relapse occurred in 36 patients (57.1%), and the treatment-related mortality was 7.9%. After a median follow up of 40.3 months (range, 12.8–83.2 months), the 3-year OS and PFS rates were 48.3 ± 6.4% and 40.1 ± 6.8%, respectively (Figure 1A). There were no differences in OS depending on histologic subtype, with the exception of anaplastic large cell lymphoma (*P* = 0.595, Figure 1B). PIT exhibited greater differentiation of survival than IPI (80.8 months in PIT 0–1, 15.0 in PIT 2, and 8.8 months in PIT 3–4, *P* = 0.011, Figure 1D). Multivariate analysis showed that performance status (≥2), visual (5-PS ≥ 4) and combined (poor responder by all three assessments) assessment in interim PET/CT scans were independent prognostic variables in PFS.

**Table 1:** **Characteristics of the patients**

Parameter	No. of patients	%
Median age in years (range)	60 (20–81)	
Histology		
PTCL-U	17	27.0
Anaplastic large cell (ALK-negative)	6 (3)	9.5
Angioimmunoblastic	10	15.9
Extranodal NK/T cells	27	42.9
Enteropathy-associated	1	1.6
Systemic cutaneous or mycosis fungoides	1	1.6
Hepatosplenic γδ	1	1.6
Stage		
I-II	24	38.1
III-IV	39	54.0
B symptoms	26	41.3
Bone marrow involvement	18	28.6
IPI		
Low/Low-intermediate	22/10	34.9/15.9
High-intermediate/High	16/15	25.4/23.8
PIT		
0/1 factor	12/22	19.0/34.9
2 factors	14	22.2
3-4 factors	15	23.8
Primary chemotherapy		
CHOP/ CHOP-like	53	84.1
Others (IMVP-16, EPOCH and VIDL)	10	15.9
Involved field radiation therapy	15	23.8
Response to primary chemotherapy		
CR/PR	34/7	54.0/11.1
SD or PD	18	28.6
Non-measurable	4	6.3
Performance of autologous stem cell transplantation	9	14.3
Treatment-related toxicity	5	7.9

*Abbreviations:* No., number; PTCL, peripheral T-cell lymphoma; IPI, international prognostic index; PIT, prognostic index for PTCL-U; CR, complete remission; PR, partial response; SD, stable disease; PD, progressive disease.

### Prognostic significance of interim PET/CT based on visual assessment

Interim PET/CT images were analyzed by a consensus of two nuclear medicine physicians who were unaware of the clinical information. The sensitivity, specificity, positive predictive value (PPV), and negative predictive value (NPV) of visual assessment (Grade ≥ 3) for the prediction of disease progression were 69.4%, 65.2%, 87.8%, and 96.1%, respectively. Seven patients showed false-positive uptake during primary chemotherapy. Five patients with nasal-type NK/T cell lymphoma continued to have significant metabolic uptakes compared with the mediastinal blood pools that were confirmed to be inflammatory lesions by loco-regional biopsy. In addition, two patients with hot uptake in the gastric and colonic regions were confirmed to show inflammatory changes by endoscopic biopsies. In contrast, three patients classified as grade 3 by visual assessment exhibited false interim PET/CT negativity; however, the original lesion was partially regressed.

**Figure 2:**
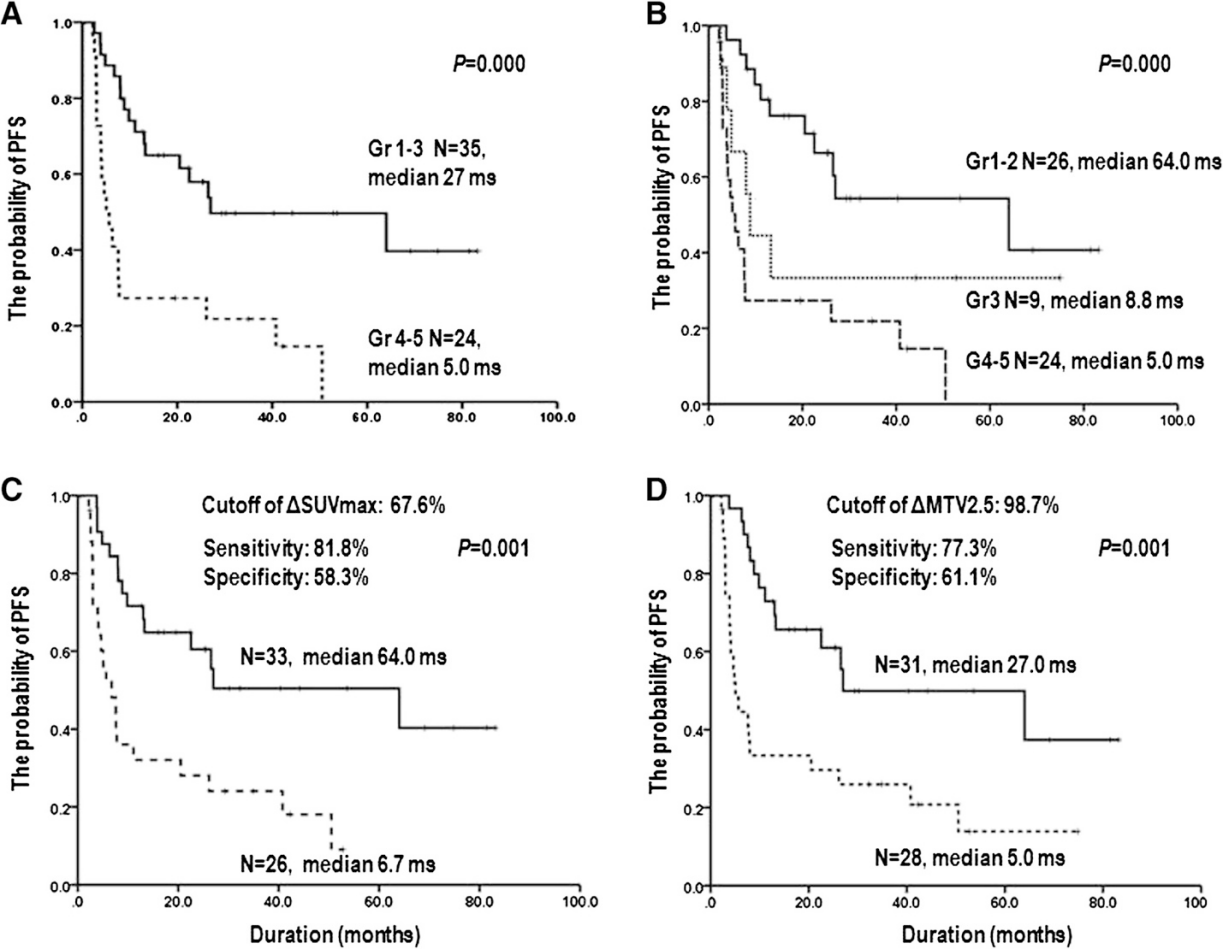
**Kaplan-Meier survival curves of PFS according to Deauville five-point scale (A, B), the SUVmax reduction rate with the optimal cutoff value of 67.6% (C), and the MTV2.5 reduction rate with the optimal cutoff value of 98.7% in interim PET/CT (D).**

Patients with interim PET/CT-positive (grade 4–5) on visual assessment showed a higher relapse rate than patients with interim PET/CT-negative (grade 1–3) (27.3% vs. 9.9%, *P* = 0.02). In addition, patients with the interim PET/CT-positive showed significantly shorter PFS times compared to patients with interim PET/CT-negative (5.0 vs. 27.0 months, *P* = 0.000, Figure 2A). However, among the 35 patients classified as interim PET/CT negative, 9 patients with grade 3 had a poor clinical outcome compared with 26 with grades 1–2 (8.8 vs. 64.0 months, *P* < 0.001; Figure 2B). A high relapse rate in patients with grade 3 or false-negative interpretation of interim PET/CT accounted for the difference in survival.

### Prognostic significance of interim PET/CT based on quantitative-assessments

In ROC analysis, the optimal cutoff values for **Δ**SUVmax and **Δ**MTV2.5 were 67.6% with an AUC of 0.673 (*P =* 0.002, 95% confidence interval (CI); 0.626–0.874) and 98.7% with an AUC of 0.627 (*P* < 0.001, 95% CI; 0.590–0.852), respectively. Each interim PET/CT assessment based on the ΔSUVmax cutoff, and ΔMTV2.5 cutoff was predictive of disease progression with relatively high positive predictive values (PPV) (92.3% and 92.8%, respectively) and negative predictive values (NPV) (90.0% and 92.8% respectively) (Table 2).

**Table 2:** **Receiver operating characteristic (ROC) values for predicting disease progression**

Assessment parameter	Sn (%)	Sp (%)	PPV (%)	NPV (%)	Area under ROC curves	95% CI	*P*value
Visual assessment (Grade ≥ 3)	69.4	65.2	87.8	96.1	0.731	0.597-0.864	0.003
ΔSUVmax cutoff	81.8	58.3	92.3	90.0	0.673	0.626-0.874	0.002
ΔMTV2.5 cutoff	77.3	61.1	92.8	93.5	0.627	0.590-0.852	0.005

*Abbreviations*: Sn, sensitivity; Sp, specificity; PPV, positive predictive value, NPV, negative predictive value, ΔSUVmax, reduction rate of the maximal standardized uptake value; ΔMTV2.5, reduction rate of metabolic tumor volume.

The response assessment of interim PET/CT based on the **Δ**SUVmax, and **Δ**MTV2.5 showed significant prognostic potential for PFS. Similarly, based on SUV-based (optimal cutoff value of 67.6%) and MTV-based assessments (optimal cutoff value of 98.7%), the probability of 3-year PFS in the 33 patients who achieved **Δ**SUVmax > the optimal cutoff was 52.6% compared with 24.7% in the 26 patients who failed to achieve the optimal cutoff (*P* = 0.001). Moreover, the 3-year PFS rate for 31 patients who achieved **Δ**MTV2.5 > the optimal cutoff was 49.9%. Furthermore, the 3-year PFS rate for the 28 patients with lower than the optimal cutoff of **Δ**MTV2.5 was 25.9% (*P* < 0.005; Figure 2C and D).

### Predictive efficacy of Interim PET/CT based on combined three parameters

**Figure 3:**
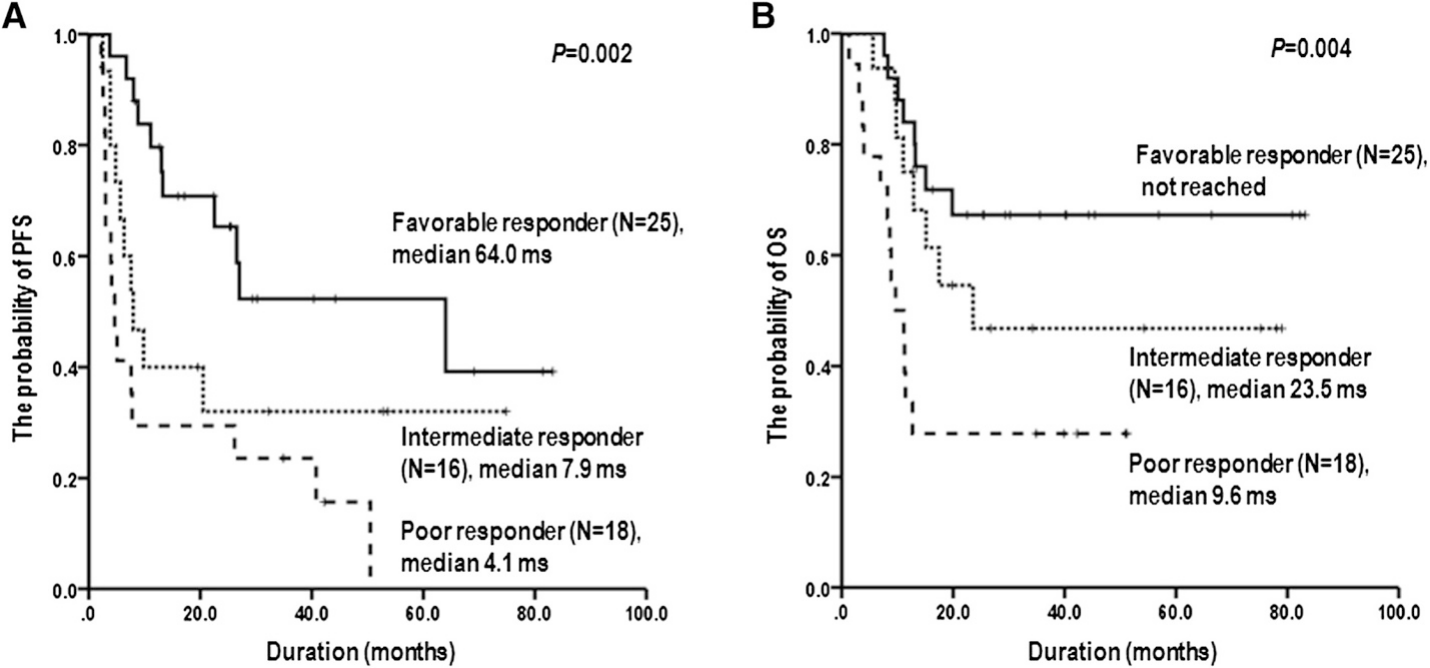
**Kaplan-Meier survival curves of PFS (A) and OS (B) according to groups classified by the three parameters and consisting of grade 4 or 5 in 5-PS, low reduction of the optimal cutoff of ΔSUVmax and ΔMTV2.5.**

Although an interim PET/CT response based on single-parameter assessment could increase the predictive power for clinical outcomes, it was unable to distinguish the outcomes in the good and poor response groups, particularly in patients with grade 3 or higher or those with less than the optimal cutoff of **Δ**SUVmax or **Δ**MTV2.5. To increase the predictive accuracy of interim PET/CT, we divided patients into three groups according to the sum of scores for the three adverse responses based on the visual, SUV-based, and MTV-based assessments. This combined assessment using the three parameters consisted of each adverse response, including grade 4 or 5 in 5-PS and little reduction in the optimal cutoffs of **Δ**SUVmax and **Δ**MTV2.5: i) favorable responders were satisfied with no adverse response assessed by the three parameters, ii) poor responders had three adverse responses, and iii) intermediate responders were satisfied with one or two adverse responses in three parameters. The clinical outcomes of patients in the favorable group were significantly superior to those of patients in the poor or intermediate responder group (*P* = 0.002, *P* = 0.004, respectively, Figure 3A, B). This classification based on the “poor” response category of visual, **Δ**SUVmax and **Δ**MTV2.5 assessments on interim PET/CT showed the subdividing ability for predicting the outcomes compared with single-parameter assessment.

## Discussion

Currently, evaluation of the interim response using PET/CT is considered predictive of early relapse and/or reduced survival in diffuse large B-cell lymphoma (DLBCL) and Hodgkin’s lymphoma (HL) [28]. In DLBCL, patients achieving interim PET negativity after two or four cycles of chemotherapy generally had more favorable outcomes compared with those who were interim PET-positive [29-31]. The prognostic role of interim PET/CT in PTCL is less-well established than that in DLBCL. One study reported that negative interim or post-therapy interim PET/CT did not associate with improved the survival In NK-T cell lymphoma [32]. However, some recent studies have suggested that the interim PET response may be also useful for predicting the outcome in PTCL. A retrospective study of mature T-cell and NK/T cell lymphoma reported that patients achieving interim PET/CT negativity showed an improved 2-year PFS and OS compared with those with interim PET/CT positivity [33]. Another retrospective study yielded similar results regarding the prognostic role of interim PET/CT [34]. However, the major drawback in these reports was the lack of uniformity or reliable criteria for interim PET interpretation.

Although visual analysis using 5-PS or quantitative imaging assessment is generally used to interpret interim PET/CT, each method has limitations if used alone to interpret interim PET/CT. Visual comparison of FDG uptake can be optically distorted due to different background levels and inter-rater discrepancy [35]. For example, a previous study reported that about 22% of the cases may have discrepant Deauville scores between the two independent readers [36]. Moreover, a study conducted in patients with HL suggested that quantitative assessment could better predict outcome than visual analysis [37]. However, the mid-treatment quantitative assessment also has several limitations, such as the definition of positivity within inter-observer interpretations, the need to consider the volumetric changes of tumors during chemotherapy, and the interpretation of minimal residual uptake at sites of physiologic anatomical FDG uptake. As the use of quantitative assessment combined with Deauville scores could be very efficient in discriminating among survival outcomes, we previously analyzed interim PET/CT using a combination of the visual, **Δ**SUVmax, and ΔMTV2.5 parameters and classified subjects into three groups: favorable, intermediate, and poor responders in DLBCL [38].

In the present study, two nuclear medicine physicians interpreted the PET/CT scans at the same time to decrease the discrepancy rate between observers. In addition, quantitative assessment with **Δ**MTV2.5 cutoff was helpful for predicting the progression during mid-therapy. Therefore, the response assessment of interim PET/CT based on the percentage of MTV reduction should be adapted to assess response early or would be helpful in increasing the predictive value combined with SUV-based assessment during the course of primary chemotherapy in PTCL. The present analysis of 59 patients suggested that each interim PET/CT response based on 5-PS, **Δ**SUVmax, and ΔMTV2.5 after three and four courses of induction treatment had predictive value for PFS in patients with PTCL; no significant difference was observed between the visual and quantitative assessments for predicting the progression.

Furthermore, the threshold value of visual assessment for distinguishing outcomes is another major concern of using interim PET/CT as a predictive marker. 4^th^ International Workshop on PET held in Menton suggested that interim PET/CT image were graded as negative or positive by comparison of initial PET/CT and grade 1–3 considered as negative and grade 4–5 considered for positive. However, this grading process is independent of the size of the residual tumor and it may be appropriate to restrict the complete metabolic response category to grades 1 and 2 in some situation [39]. In the current study, patients with grade 3 experienced poor clinical outcomes compared with patients with grade 1 and 2. The different clinical outcomes resulted from high relapse rate or false negative uptakes in patients with grade 3. The definition of positivity using grade 1–2 reported relatively low PPV with high NPV compared to quantitative assessments in ROC analysis. Low PPV could make it difficult to modify the therapeutic plans with interim PET-adapted dose escalation or high-dose chemotherapy in poor responders. Therefore, it was reported that the combined assessment using the three parameters was identified to be more predictive of survival outcomes compared with single-parameter assessment, and this combined approach may help to overcome the limitations of interim PET/CT evaluation.

## Conclusions

Visual, quantitative SUV-based, and MTV-based assessment in interim PET/CT showed prognostic significance for mid-treatment response analysis in patients with PTCL, and the combined approach using the three parameters could have more predictive accuracy for disease progression between patients with different survival outcomes compared with single-parameter assessment.
